# Molecular Evolution of Sunflower and Cottonseed Oils During Preparation of Uzbek Palov

**DOI:** 10.3390/foods15183222

**Published:** 2026-09-11

**Authors:** Umrbek Mavlanov, Sharofiddin Nuriddinov, Soyibjon Bozorov, Ergashev Oybek, Kodirov Olimjon, Meliboyev Mirazam, Kakharova Mukharram, Sardorbek Rahimovich, Sardorjon Shukurov Salimovich, Suvanqul Ravshanov, Sherzod Kurambayev, Sarvar A. Kakhkhorov, Tomasz Paweł Czaja, Dilbar Dalimova, Bekzod Khakimov

**Affiliations:** 1 Department of Food Science, University of Copenhagen, Rolighedsvej 26, 1958 Frederiksberg C, Denmark; sarvar@food.ku.dk (S.A.K.); tomasz.czaja@food.ku.dk (T.P.C.); 2 Center for Advanced Technologies, Talabalar Shaharchasi 3A, Tashkent 100174, Uzbekistan; ssharofnur@gmail.com (S.N.); soyibjon0312@gmail.com (S.B.); sardorshukurov15@gmail.com (S.S.S.); dilbar.dalimova@gmail.com (D.D.); 3 Department of Food Technology, Namangan State Technical University, I. Karimov Street 12, Namangan City 160103, Uzbekistan; okergashev711@gmail.com (E.O.); olimjonqodirov18@gmail.com (K.O.); mirzamm@bk.ru (M.M.); kaxarovamuxarram@gmail.com (K.M.); 4 Department of Physical Chemistry, National University of Uzbekistan Named After Mirzo Ulugbek, University Str. 4, Tashkent 100174, Uzbekistan; otajonovsardor1994@gmail.com; 5 Department of Biotechnology, Tashkent Institute of Chemical Technology, Navoiy Street 32, Tashkent 100011, Uzbekistan; svankulravshanov@gmail.com; 6 Department of Food Technology, Urgench State University Named After Abu Rayhan Biruni, Kh. Alimjon 14, Urgench City 220100, Uzbekistan; skurambayev@list.ru

**Keywords:** Foodomics, edible oils, Uzbek Palov, food–oil interactions, lipid oxidation, FTIR, benchtop NMR, high-field NMR

## Abstract

Understanding how vegetable-oil composition evolves during food preparation is essential for evaluating food quality and nutritional value. Palov is Uzbekistan’s most widely consumed and culturally significant dish, serving as an everyday meal and a central element of hospitality and communal traditions. We hypothesised that food–oil interactions during Palov preparation direct molecular changes that cannot be reproduced by controlled heating alone. Two independent batches of Palov were prepared with sunflower or cottonseed oil under household cooking conditions. Oils were sampled at six stages and compared with heating controls conducted under closely matched temperature–time profiles without food ingredients. Samples were analysed using Fourier-transform infrared spectroscopy, benchtop and high-field ^1^H nuclear magnetic resonance spectroscopy. Cooking caused substantially greater fatty-acid changes than heating alone. In cottonseed oil, polyunsaturated fatty acids decreased from approximately 44 to 32 mol%, monounsaturated fatty acids increased from approximately 19 to 26 mol%, saturated fatty acids increased from approximately 36.5 to 41.5 mol%, and *trans*-fatty acids increased from approximately 0 to 2%. Heating alone produced greater accumulation of conjugated dienes and hydroperoxides, whereas cooking produced more dynamic changes in sterol-, aldehyde- and glyceride-related signals. These findings demonstrate that food–oil interactions redirect fatty-acid composition, hydrolysis and oxidation-product evolution rather than merely altering the extent of thermal damage. Realistic culinary models therefore provide a more representative description of edible-oil transformation than heating-only experiments.

## 1. Introduction

Edible oils are fundamental to domestic and industrial food preparation, serving as heat-transfer media, sources of essential fatty acids, and carriers of lipid-soluble vitamins and flavour compounds. Their molecular composition influences the nutritional value, sensory quality [1], oxidative stability and safety of cooked foods. During cooking, oils undergo interconnected oxidation, hydrolysis, isomerisation and polymerisation reactions that modify both major fatty acids and minor lipid constituents [2]. Understanding these transformations under realistic culinary conditions is therefore essential for evaluating oil quality and food safety [3]. Polyunsaturated fatty acids are particularly susceptible to radical-mediated oxidation, producing hydroperoxides that subsequently decompose into aldehydes, ketones, alcohols, acids, epoxides and other secondary oxidation products. Heating of sunflower oil causes progressive depletion of unsaturated acyl groups, together with the formation of multiple oxidation products [4]. Subsequent NMR and FTIR studies showed that hydroperoxides, aldehydes, epoxides, *cis* double bonds and carbonyl compounds evolve through coordinated degradation pathways rather than as independent reactions [5,6,7]. Frying also generates monoepoxy fatty acids [8], while oil composition, food type and frying frequency influence the formation of polar compounds, volatile products and *trans*-fatty acids [9,10,11].

Most mechanistic studies, however, heat oils without food or investigate repeated frying of a single food [12,13,14]. Real cooking with multiple ingredients is considerably more complex because oils continuously interact with water, proteins, carbohydrates, minerals, phospholipids, antioxidants, pro-oxidants and food-derived lipids. These interactions influence hydrolysis, lipid migration, oxidation, carbonyl chemistry and partitioning of degradation products, making food an active determinant of oil chemistry. Frying vegetables, fish, meat and carbohydrate-rich foods produces distinct oxidation profiles under comparable conditions [15]. Potato frying, for example, promotes greater accumulation of total polar compounds and polymerised triacylglycerols, whereas chicken frying generates higher concentrations of epoxy-, keto- and hydroxy-fatty acids [15]. More targeted studies show that food constituents modify specific pathways: reactive aldehydes partition differently between oil and fried foods [16], amino acids alter formation and distribution of glycerol-core and volatile aldehydes, and multiomics analysis indicates that chicken promotes greater lipid hydrolysis, whereas French fries favour oxidative degradation and oxidised triacylglycerol formation [17].

Despite these advances, much less is known about multicomponent dishes prepared through sequential ingredient addition under continuously changing temperature, moisture and oxygen conditions. Direct comparisons between realistic cooking and heating-only controls exposed to closely matched temperature histories also remain scarce, limiting the ability to distinguish heat-driven transformations from those arising through food–oil interactions. Addressing this challenge requires analytical approaches capable of characterising several molecular classes simultaneously. FTIR spectroscopy detects changes associated with unsaturation and *cis–trans* isomerisation, whereas high-field ^1^H NMR quantifies fatty acid groups, oxidation products, sterols and partial glycerides. Benchtop ^1^H NMR has been validated against high-field NMR for fatty-acid quantification [18], and its complementary use with FTIR has been demonstrated for edible-oil characterisation [19]. Integrating these platforms follows the Foodomics concept of studying food as an interconnected molecular system rather than through isolated quality indicators [20].

The present study investigated these interactions during preparation of Palov, Uzbekistan’s most widely prepared and consumed traditional dish and an element of UNESCO’s Representative List of the Intangible Cultural Heritage of Humanity [21]. Palov is served as an everyday meal and at weddings, religious celebrations and other major social events. Sequential cooking of oil with meat, onions, carrots, water, and rice creates a dynamic multiphase system in which lipids continuously interact with animal and plant tissues, moisture, starch, and endogenous metabolites. Its cultural importance and chemically evolving cooking environment make Palov a highly relevant model for investigating edible-oil chemistry under realistic culinary conditions. We hypothesised that food–oil interactions during Palov preparation direct fatty-acid alterations, oxidation, hydrolysis and transformation of minor lipid constituents, producing molecular changes that cannot be reproduced by heat exposure alone. To test this hypothesis, sunflower and cottonseed oils collected throughout Palov preparation were compared with the same oils subjected to heating-only experiments under closely matched temperature profiles. An integrated Foodomics approach combining FTIR with benchtop and high-field ^1^H NMR spectroscopy was used to distinguish heat-driven transformations from those arising through interactions with the food matrix.

## 2. Materials and Methods

### 2.1. Chemicals and Materials

Deuterated chloroform (CDCl_3_, 99.8 atom% D) containing 0.03% (*v*/*v*) tetramethylsilane (TMS), toluene (as IS), and all other reagents and standards were purchased from Sigma-Aldrich (Søborg, Denmark). Commercial refined sunflower and cottonseed oils were purchased from local supermarkets in Uzbekistan and stored in their original sealed containers until use.

### 2.2. Experimental Design and Sample Collections

**Figure 1:**
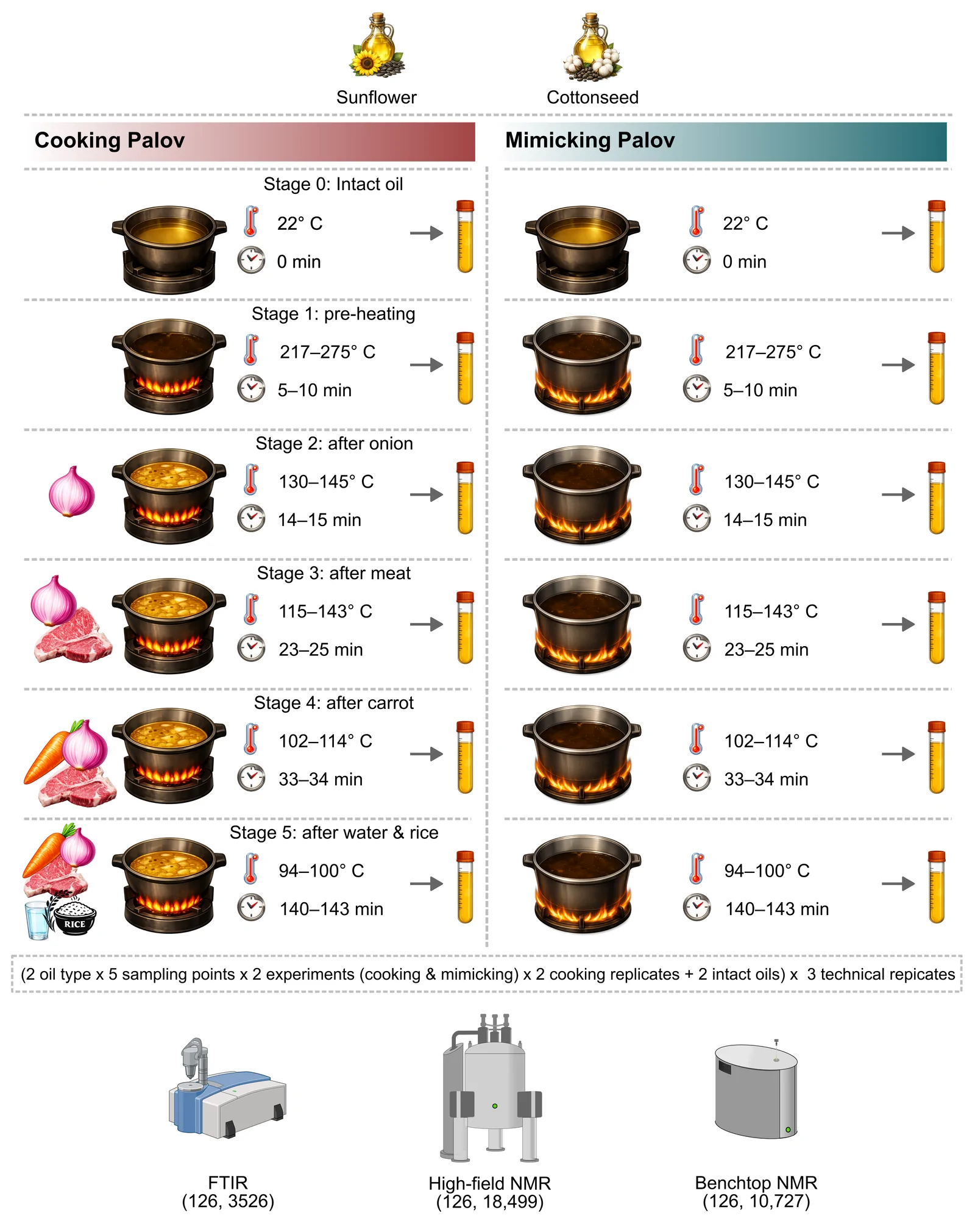
Experimental design of the Palov cooking and mimicking experiments. Sunflower and cottonseed oils were studied under two conditions: real Palov cooking in the presence of food ingredients and mimicking, in which the oils were heated without food using the same temperature and time conditions. Oil samples were collected at six stages. Two independent experimental replicates were performed for each oil and condition. This design produced 42 independent oil samples. Each sample was measured in technical triplicate by FTIR, benchtop, and high-field ^1^H NMR spectroscopy, resulting in 126 spectra per analytical platform. The numbers shown below each instrument represent the dimensions of the resulting data matrix (spectra × spectral variables).

The study compared molecular changes occurring in oils during preparation of Uzbek Palov with those produced by matched heating in the absence of food ingredients (Figure 1). Sunflower and cottonseed oils were examined under two conditions: Palov cooking and heating control. Two independent experiments were conducted for each oil and condition. During Palov preparation, onion, meat, carrot, water, and rice were added sequentially according to the traditional procedure. Oil was sampled at six predefined stages: fresh unheated oil at approximately 22 °C (Stage 0); after oil preheating (Stage 1); after cooking with onion (Stage 2); after cooking with meat (Stage 3); after cooking with carrot (Stage 4); and after addition of water and rice, immediately before serving (Stage 5). Sampling times and measured temperature ranges are presented in Figure 1. Heating-control experiments were performed without food ingredients using the same oils, heating stages, target temperatures, sampling times, and total duration as closely as technically possible. Small temperature differences were unavoidable because food addition altered thermal mass, heat transfer, and temperature recovery. At each stage, approximately 2 mL of the visible oil phase was collected using a glass pipette, cooled to room temperature, and centrifuged at 3000 rpm for 2 min at ambient temperature to separate residual water and suspended food particles before spectroscopic analysis. The separated oil phase was then transferred to 2 mL Eppendorf tubes and stored at −20 °C until spectroscopic analysis. Each sample was measured in triplicate by Fourier-transform infrared (FTIR), benchtop ^1^H NMR, and high-field ^1^H NMR spectroscopy. Independent cooking and heating experiments were treated as experimental replicates, whereas repeated instrumental measurements were treated as analytical replicates.

### 2.3. Fourier-Transform Infrared Spectroscopy

FTIR spectra were acquired using a Bruker Vertex 80 spectrometer (Bruker Optics, Ettlingen, Germany) equipped with a single-reflection diamond attenuated total reflectance accessory, a deuterated triglycine sulfate detector and a potassium bromide beam splitter. Approximately 10 μL of oil was placed on the ATR crystal maintained at 60 °C. Spectra were recorded from 4000 to 600 cm^−1^ at 4 cm^−1^ resolution using 128 co-added scans. Background spectra were recorded regularly, and the crystal was cleaned with ethanol and dried between measurements. Total *trans*-fatty acids were estimated from the negative second-derivative intensity at 966 cm^−1^ using the validated FTIR calibration described by Mavlanov et al. [19].

### 2.4. Benchtop ^1^H NMR Spectroscopy

Oil (250 μL) was mixed with 400 μL of CDCl_3_ containing 0.03% (*v*/*v*) TMS and 0.1% (*w*/*w*) toluene as the internal standard (IS), and then transferred to a 5 mm NMR tube. One-dimensional ^1^H NMR spectra were acquired using a Spinsolve 80 Ultra benchtop spectrometer with ^13^C decoupling (Magritek, Aachen, Germany), controlled by Spinsolve software version 2.6.8. Automatic sample-specific shimming was performed before acquisition. Spectra were collected using a 90° proton pulse, 32 scans, a 3.2 s acquisition time, and a 10 s relaxation delay. Fourier transformation, phase correction, and baseline correction were performed in Spinsolve. Spectra were referenced to TMS at 0.00 ppm and restricted to −0.2–10.0 ppm.

### 2.5. Determination of Fatty-Acid Composition

Relative saturated (SFA), monounsaturated (MUFA) and polyunsaturated fatty acids (PUFA) were determined from the benchtop ^1^H NMR spectra using the validated direct signal-integration method of Mavlanov et al. [18]. Briefly, PUFAs were calculated from the bis-allylic proton region relative to the α-methylene proton region, total unsaturated fatty acids from the allylic proton region, MUFAs as the difference between total unsaturated fatty acids and PUFAs, and SFAs as the remaining fatty-acid fraction. The integration regions were 1.85–2.23 ppm for allylic protons, 2.28–2.36 ppm for α-methylene protons adjacent to the carbonyl group, and 2.60–2.95 ppm for bis-allylic protons. Fatty-acid classes were expressed as percentages of total fatty acids.

### 2.6. High-Field ^1^H NMR Spectroscopy

The samples prepared for benchtop NMR were analysed without further preparation using a Bruker Avance III 500 MHz spectrometer (Bruker BioSpin, Rheinstetten, Germany) equipped with a 5 mm broadband inverse probe and SampleJet sample changer. Samples were equilibrated in the probe for 5 min and measured at 300 ± 1 K. Automated locking, tuning, matching, and shimming were performed using TopSpin 3.5 PL6 and iconNMR. Spectra were acquired with 32 scans after four dummy scans, 64 k data points, a 20 ppm spectral width, a 2.73 s acquisition time, a 4.0 s relaxation delay, a 0.01 s mixing time, and a fixed receiver gain of 11.3. Free induction decays were Fourier-transformed using 0.3 Hz line broadening, followed by phase and baseline correction. Spectra were referenced to TMS at 0.00 ppm and restricted to −0.2–10.0 ppm. ^1^H NMR spectra were globally aligned using the Icoshift algorithm [22]. Peak areas were calculated by trapezoidal numerical integration over predefined spectral regions using custom Python scripts; integration boundaries and signal assignments are provided in Table S1. Each peak area was normalised to the IS (toluene) signal in the corresponding spectrum. The resulting normalised matrix was used for subsequent statistical and chemometric analyses.

### 2.7. Statistical and Chemometric Analyses

Data processing and statistical analyses were performed using custom scripts in Python version 3.12. FTIR spectra were restricted to 3050–2800 and 1800–600 cm^−1^, while the non-informative 2800–1800 cm^−1^ region was excluded. Spectra were preprocessed using a Savitzky–Golay second derivative with a 15-point window and second-order polynomial before principal component analysis (PCA). PCA score plots were used to evaluate stage-dependent molecular trajectories, and loading plots were examined to identify the spectral regions responsible for sample separation. Targeted FTIR and NMR variables were expressed as standardised differences and visualised as heatmaps. Pairwise associations among quantified variables were assessed using Pearson correlation coefficients. Correlation matrices were calculated separately for each oil and experimental condition and displayed as lower-triangular heatmaps. Analytical replicates were averaged before statistical analysis, and independent experiments were retained as the experimental units.

## 3. Results and Discussion

### 3.1. FTIR Reveals Food-Driven Molecular Evolution of Edible Oils During Palov Preparation

To determine whether food altered oil chemistry beyond thermal exposure alone, FTIR spectra obtained during Palov preparation were compared with those of the same oils heated without food under closely matched temperature–time conditions (Figure 1 and Figure S1). Despite comparable thermal histories, the PCA score and loading patterns demonstrated that food–oil interactions continuously redirected the molecular transformation of both sunflower and cottonseed oils.

**Figure 2:**
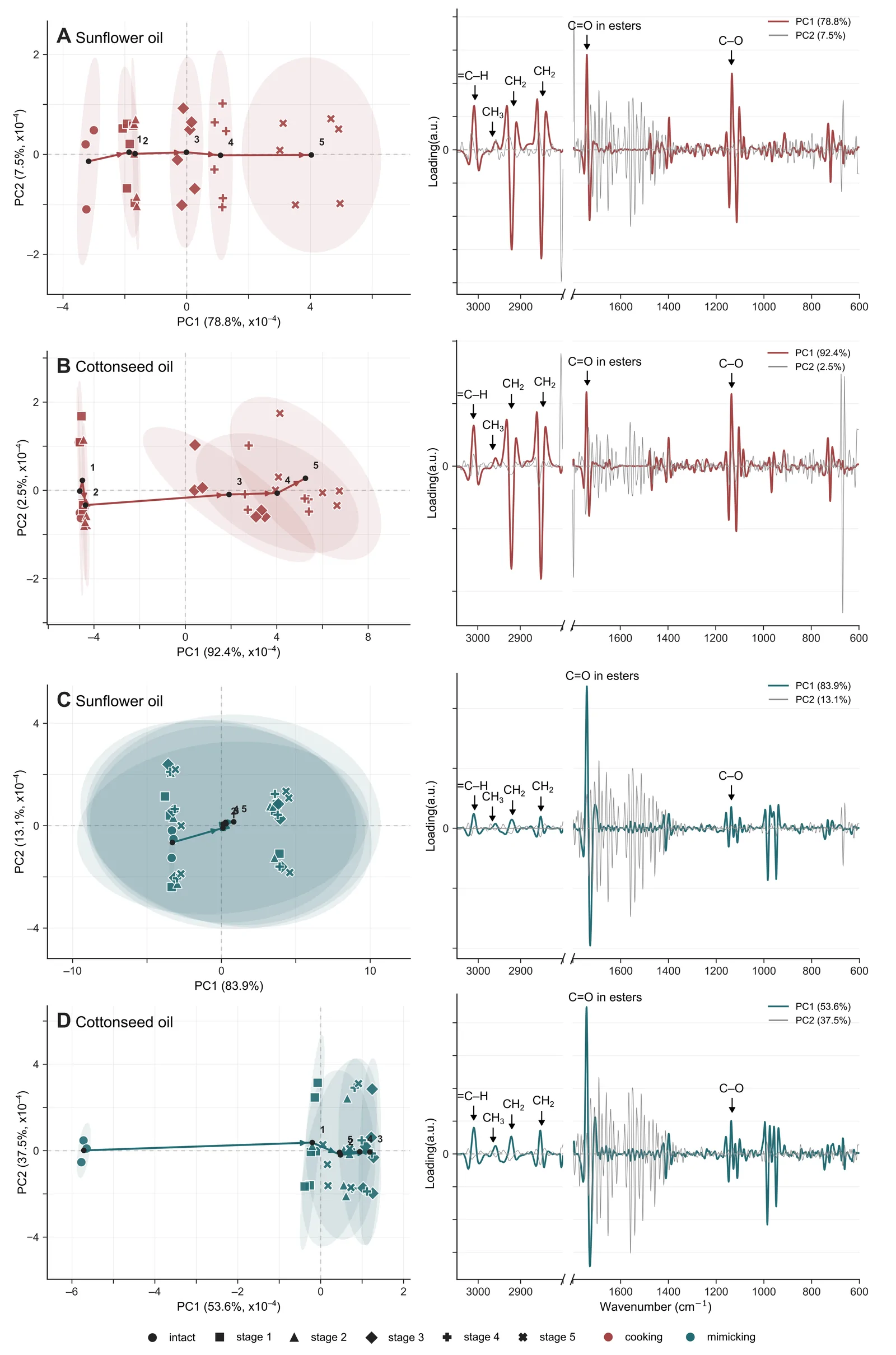
Principal component analysis (PCA) of ATR–FTIR spectra of sunflower and cottonseed oils during cooking and equivalent heating without food (mimicking). Separate PCA models were constructed for (**A**) sunflower and (**B**) cottonseed oils during cooking, and (**C**) sunflower and (**D**) cottonseed oils during mimicking. Score plots are shown on the left, with the corresponding PC1 and PC2 loading profiles on the right. Marker shapes indicate processing stages; lines connect stage centroids in chronological order; arrows show the direction of processing. Spectra were restricted to 3050–2800 and 1800–600 cm^−1^ and subjected to Savitzky–Golay second-derivative preprocessing followed by mean centring before PCA.

For sunflower oil, PCA revealed a clear stage-dependent progression during Palov preparation (Figure 2A). Each successive cooking stage shifted further from the intact oil, showing that molecular changes continued after the initial high-temperature preheating step. In contrast, during mimicking, samples from experimental runs 1 and 2 were separated mainly along PC1, whereas the processing stages within each run substantially overlapped (Figure 2C). This run-dependent separation suggests that differences between the independent heating experiments contributed more strongly to the spectral variation than the changes occurring across the later heating stages. Such differences may arise from small variations in the time–temperature profile, sample handling, or instrumental measurement. Nevertheless, the limited stage-wise movement within each run indicates that most heat-driven changes occurred during the initial preheating step, with comparatively little additional modification during prolonged heating.

The corresponding loading plots connected these score patterns to coordinated changes across several lipid-related FTIR regions. Variation at 3008 cm^−1^, assigned to *cis* olefinic =C–H stretching, indicates modification of unsaturated acyl chains. Changes at 2917 and 2850 cm^−1^ reflect alterations in asymmetric and symmetric CH_2_ stretching and therefore in the abundance, conformation, or packing of hydrocarbon chains. The contribution near 1743 cm^−1^ indicates changes in the ester carbonyl environment of triacylglycerols, while bands in the fingerprint region, including CH_2_ deformation and C–O stretching vibrations, reflect restructuring of glycerol ester bonds and the broader triacylglycerol matrix. These coordinated changes suggest that sunflower oil underwent simultaneous modification of fatty-acid unsaturation, acyl-chain organisation, and glyceride structure during cooking, whereas heating alone produced relatively little further spectral evolution after the initial thermal treatment.

Cottonseed oil exhibited the same general contrast, but the cooking-related progression was more pronounced. Successive stages moved continuously away from the intact oil, with clear separation between intermediate and final stages (Figure 2B), indicating sustained chemical transformation throughout Palov preparation. Heating without food generated a smaller stage-to-stage progression, although the samples were more dispersed than those of heated sunflower oil (Figure 2D). The corresponding loadings again identified *cis*-unsaturation bands, aliphatic CH_2_ vibrations, ester carbonyls, and glyceride-related fingerprint bands as the principal contributors. Their persistent contribution during cooking indicates continuing modification of unsaturated acyl groups and the triacylglycerol environment. The stronger response of cottonseed oil may reflect differences in its initial fatty-acid profile and minor constituents, which influence susceptibility to oxidation and other thermally induced transformations.

These FTIR patterns agree with controlled heating studies showing that edible-oil oxidation involves concurrent changes in *cis* double bonds, carbonyl environments, aliphatic chains, and glyceride-related vibrations [6,23]. Under heating alone, the rapid initial displacement followed by partial stabilisation is consistent with overlapping reaction kinetics: primary products may accumulate early and subsequently decompose, while losses of unsaturated structures may be partly balanced by the formation of new oxygenated functional groups. FTIR therefore captures the net molecular state of the oil rather than the concentration of a single oxidation product.

The continued displacement during cooking shows that food did not merely increase or decrease the overall extent of thermal deterioration. Sequential addition of onion, meat, carrot, water, and rice repeatedly changed the oil phase through moisture release, lipid exchange, hydrolysis, altered oxygen transfer, and the introduction of food-derived antioxidants, pro-oxidants, proteins, and other reactive constituents. These changes prevented the oil from following the comparatively simple, predominantly temperature-driven progression observed during heating alone.

This interpretation is consistent with frying studies showing that food composition determines the relative formation of polymerised triacylglycerols, oxidised fatty acids, and other polar compounds within the same frying medium [15]. Oxidative lipidomics has likewise demonstrated that chicken promotes greater lipid hydrolysis, whereas French fries favour oxidative degradation and oxidised triacylglycerol formation [17]. Food matrices also influence the partitioning and subsequent reactions of lipid-derived aldehydes [16,24]. The present study extends this knowledge from single-food frying systems to a multicomponent dish, demonstrating through global FTIR fingerprints that sequential food–oil interactions continuously redirect oil chemistry throughout cooking.

Overall, Figure 2 shows that heat initiated the first major molecular shift, whereas food addition sustained and redirected subsequent changes in fatty-acid unsaturation, hydrocarbon-chain organisation, ester carbonyl environments, and glyceride structure. Thus, Palov preparation generated a continuing, food-driven molecular progression that could not be reproduced by matched heating of the isolated oils.

### 3.2. Food–Oil Interactions Reshape Fatty Acid Composition and Trans-Fatty Acid Formation

**Figure 3:**
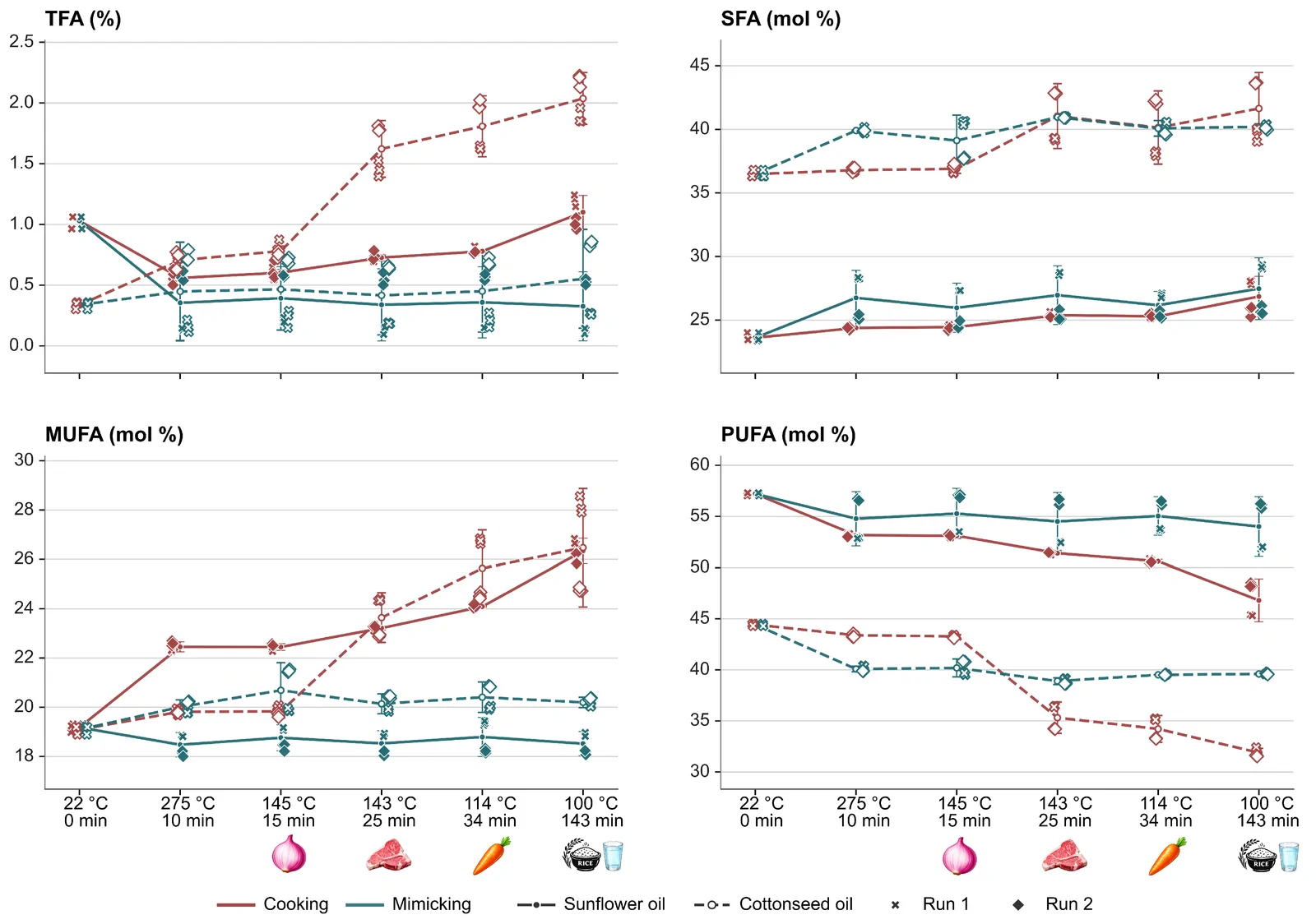
Changes in fatty-acid composition of sunflower and cottonseed oils during Palov cooking and equivalent heating without food (mimicking). Saturated fatty acids (SFAs), monounsaturated fatty acids (MUFAs), and polyunsaturated fatty acids (PUFAs) were calculated from benchtop ^1^H NMR spectra, whereas *trans*-fatty acids (TFAs) were determined from ATR–FTIR spectra. Crosses and diamonds represent individual measurements from experimental runs 1 and 2, respectively. Ingredient symbols indicate the addition of onion, meat, carrot, rice, and water during cooking. SFAs, MUFAs, and PUFAs are expressed as mole percentages (mol%), calculated as the number of moles of fatty acids within each class divided by the total number of moles of quantified fatty acids and multiplied by 100. Thus, mol% represents the relative molecular proportion of each fatty-acid class rather than its proportion by mass.

The global FTIR changes described earlier were accompanied by pronounced alteration of fatty acid composition (Figure 3). Saturated fatty acids, monounsaturated fatty acids, and polyunsaturated fatty acids were quantified by benchtop ^1^H NMR, whereas total *trans*-fatty acids were estimated from FTIR spectra. Although both cooking and matched heating (mimicking) altered the oils, Palov preparation generally produced larger and more sustained changes, particularly during the later stages.

In sunflower oil, cooking increased SFAs from approximately 24 to 27 mol% and MUFAs from approximately 19 to 26 mol%, while PUFAs decreased from approximately 57 to 47 mol% (Figure 3). The most pronounced divergence from heating alone occurred after meat addition and continued through the carrot and water/rice stages. Under matched heating, SFAs increased moderately, but MUFAs remained close to 19 mol% and PUFAs decreased only slightly to approximately 54 mol%. Thus, the substantial MUFA enrichment and larger PUFA depletion observed during cooking cannot be explained by thermal exposure alone. These compositional shifts provide targeted molecular support for the continuous stage-dependent FTIR progression observed in Figure 2A, whereas the smaller changes during heating are consistent with the greater overlap of later stages in Figure 2C.

Cottonseed oil exhibited even stronger food-dependent alterations. During Palov preparation, PUFAs decreased from approximately 44 to 32 mol%, while MUFAs increased from approximately 19 to 26 mol%, and SFAs from approximately 36.5 to 41.5 mol% (Figure 3). Heating alone produced smaller changes: PUFAs declined to approximately 39–40 mol%, MUFAs remained near 20 mol%, and SFAs increased to approximately 40 mol%. The divergence became especially evident after meat addition, when cooking produced a sharp decrease in PUFAs, together with increasing MUFAs and SFAs. This marked compositional progression is consistent with the stronger separation among cottonseed-oil cooking stages in Figure 2B.

Preferential PUFA depletion is chemically consistent with their greater susceptibility to radical-mediated oxidation because bis-allylic hydrogen atoms are readily abstracted [2,3]. However, selective oxidation alone is unlikely to account for the simultaneous increase in MUFAs and SFAs, particularly because fatty-acid classes are expressed as relative proportions. During cooking, oil is absorbed by food while lipids from meat and other ingredients migrate into the oil phase. Because animal lipids are generally richer in SFAs and MUFAs than sunflower and cottonseed oils, bidirectional lipid transfer could contribute to both the relative enrichment of SFAs and MUFAs and the dilution of vegetable-oil PUFAs. Previous frying studies have similarly shown that the final fatty-acid profile of an oil reflects the combined effects of selective oxidation, oil uptake by food, and migration of food-derived lipids into the frying medium [9,17].

The TFA profiles provided an additional distinction between cooking and heating (Figure 3). In sunflower oil, TFAs remained comparatively low after the initial oil measurement, although cooking produced a late increase to approximately 1.1%, compared with approximately 0.3% after matched heating. Cottonseed oil showed a much stronger response: TFAs increased progressively during cooking, particularly after meat addition, and reached approximately 2.0% at the final stage, whereas the heating control remained near 0.4–0.6%. Therefore, the largest *trans*-isomerisation response occurred specifically when cottonseed oil interacted with the Palov ingredients.

Thermally induced TFA formation is generally attributed to radical-mediated *cis–trans*-isomerisation, which is promoted by temperature, heating duration, and oxidative stress. The substantially greater increase during cottonseed-oil cooking suggests that the food matrix modified the reaction environment beyond the temperature effect. Haem compounds, transition metals, and reactive species introduced by meat may promote hydrogen abstraction and radical propagation, while changes in moisture, oxygen availability, and antioxidant content may influence competition between oxidation and isomerisation. These mechanisms were not measured directly and should therefore be considered chemically plausible interpretations rather than demonstrated pathways.

Overall, Palov preparation caused greater fatty-acid alterations than matched heating, with the clearest effects being PUFA depletion, MUFA enrichment, and enhanced TFA formation in cottonseed oil. These results indicate that food–oil interactions reshape fatty-acid composition through the combined effects of selective oxidation, bidirectional lipid transfer, and thermally promoted isomerisation, providing targeted chemical evidence for the global molecular progression identified by FTIR in Section 3.1.

### 3.3. Food Redirects Sterol Transformation, Glyceride Hydrolysis and Oxidation Pathways During Cooking

**Figure 4:**
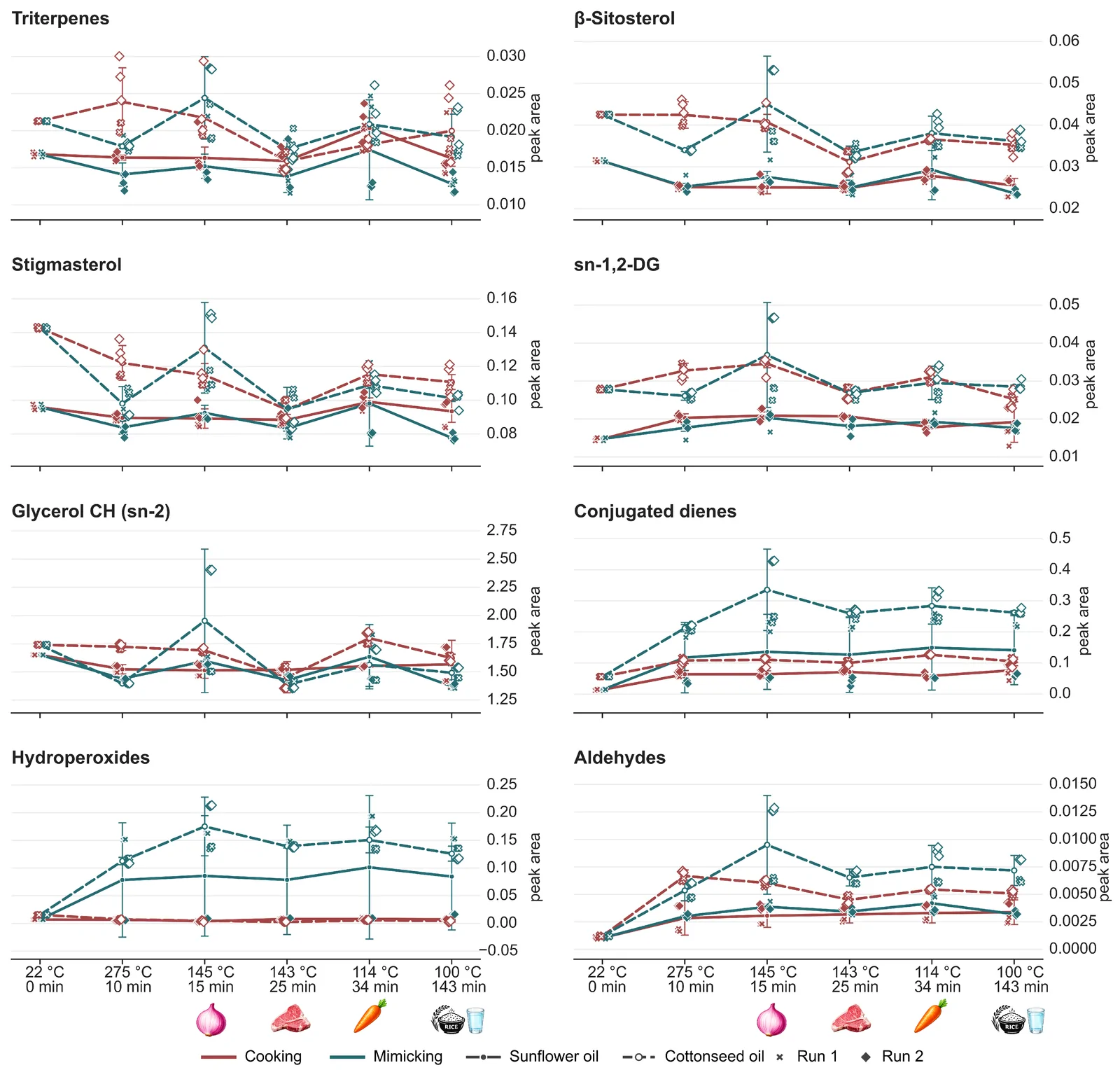
Changes in minor lipid compounds and oxidation products in sunflower and cottonseed oils during Palov cooking and equivalent heating without food (mimicking). Triterpenes, β-sitosterol, stigmasterol, *sn*-1,2-diglycerides (*sn*-1,2-DG), the glycerol CH signal at the *sn*-2 position, conjugated dienes, hydroperoxides, and aldehydes were quantified using high-field ^1^H NMR spectroscopy. Values are expressed as integrated NMR signal areas, normalised to the toluene internal standard. Crosses and diamonds represent individual measurements from experimental runs 1 and 2, respectively. Ingredient symbols indicate the addition of onion, meat, carrot, rice, and water during Palov cooking.

The fatty-acid alterations described above were accompanied by stage-dependent changes in minor lipid constituents, partial glycerides, glycerol-backbone resonances and oxidation products (Figure 4). These responses differed between cooking and matched heating, and were often non-monotonic, indicating that the recovered oil reflected concurrent formation, degradation, migration and partitioning processes rather than cumulative thermal deterioration alone.

Sterol-related signals responded differently according to oil type, treatment and cooking stage. In sunflower oil, β-sitosterol and stigmasterol decreased after preheating and remained below their initial levels, with a further decline at the final cooking stage. Cottonseed oil showed larger but less uniform fluctuations, including temporary increases during heating and subsequent decreases during cooking. Triterpene signals also varied rather than following a simple monotonic loss. These profiles are compatible with several simultaneous processes. Phytosterols are susceptible to thermal and oxidative degradation, producing oxidation products that are no longer represented by the intact sterol resonances measured here [25]. However, concentration changes in recovered cooking oil may additionally reflect migration between oil and food, dilution by food-derived lipids, and partitioning into protein- or starch-rich phases. The data therefore support dynamic sterol transformation and redistribution, but not uniform progressive degradation across all stages and oils.

The partial glycerides provided direct evidence that hydrolysis contributed to oil remodelling. Signals assigned to sn-1,2-diglycerides changed throughout both experiments, but cooking produced more stage-dependent and oil-specific behaviour, particularly in cottonseed oil after onion addition and during the subsequent meat, carrot and water/rice stages. Diglycerides arise mainly through cleavage of triacylglycerol ester bonds, although they may also undergo acyl migration, oxidation, re-esterification and polymerisation during heating. Their concentrations therefore represent the balance between formation and further conversion rather than the cumulative extent of hydrolysis. The non-linear profiles observed here are consistent with a continuously changing reaction environment in which hydrolysis, acyl migration and lipid exchange occurred simultaneously.

Hydrolysis was probably favoured by the progressive development of oil–water interfaces during cooking [26]. Onion, meat and carrot released moisture before the deliberate addition of water for rice cooking, substantially increasing contact between triacylglycerols and the aqueous phase. Such interfacial conditions promote ester-bond cleavage and formation of diglycerides, monoglycerides and free fatty acids. The accompanying changes in glycerol CH resonances further indicate modification of the triacylglycerol framework. These signals decreased after preheating, increased at selected ingredient-addition stages and changed again towards the end of cooking, particularly in cottonseed oil. Their coordinated behaviour with the diglyceride signals is consistent with hydrolysis and glyceride restructuring, although lipid migration from meat and oil uptake by the food may also have contributed. Similar acylglycerol changes have been described in frying oils, where hydrolysis, oxidation and reorganisation of partial glycerides proceed concurrently.

Hydrolysis should not be interpreted independently of oxidation. Partial glycerides and free fatty acids increase oil polarity and modify interfacial organisation, which can influence oxygen transfer and the accessibility of unsaturated acyl chains to reactive species. Conversely, oxidation can destabilise glyceride structures and facilitate further degradation. The increasingly heterogeneous Palov system, containing aqueous droplets, suspended food particles, endogenous food lipids, and polar metabolites, would therefore be expected to couple hydrolytic and oxidative reactions more strongly than dry heating of oil alone.

This coupling was evident in the oxidation-product profiles (Figure 4). Heating without food generally produced substantially greater accumulation of conjugated dienes and hydroperoxides, particularly in cottonseed oil. In the heating controls, both primary oxidation markers increased rapidly during the early stages and remained elevated thereafter. During cooking, conjugated dienes increased more modestly, while hydroperoxide signals remained close to their initial levels. Because conjugated dienes and hydroperoxides are early products of PUFA oxidation, these differences show that the food-containing system did not follow the same primary oxidation pattern as the isolated oil exposed to a similar temperature history.

Lower hydroperoxide abundance during cooking should not, however, be interpreted as evidence of negligible oxidation. Hydroperoxides are unstable intermediates that may decompose rapidly into aldehydes, ketones, alcohols and shorter-chain products at elevated temperatures. They may also partition into the food phase or undergo reactions promoted by food-derived metals, proteins and other reactive constituents. The measured values therefore represent the balance between hydroperoxide formation, decomposition, transfer, and secondary reaction.

Aldehyde profiles supported this more complex interpretation. In sunflower oil, cooking and heating produced broadly similar increases, with only modest treatment-related differences. In cottonseed oil, cooking generated a pronounced aldehyde increase during preheating, whereas the heating control showed higher values at several later stages (Figure 4). Thus, cooking did not uniformly increase or decrease aldehyde accumulation; instead, it altered their temporal formation and fate. Lipid-derived aldehydes can react with amino acids, peptides, and proteins through Schiff-base formation, Michael addition, and related carbonyl reactions. During Palov preparation, aldehydes may therefore have been consumed by reactions with meat-derived proteins or Maillard intermediates or transferred into the food rather than remaining in the recovered oil. Food-dependent partitioning of reactive aldehydes and amino-acid-mediated modification of aldehyde formation have been demonstrated in frying systems.

The evolving antioxidant and pro-oxidant environment provides an additional explanation for the divergence between cooking and heating. Onion and carrot may release phenolic compounds, carotenoids and other reducing constituents capable of transiently limiting radical propagation. Conversely, meat supplies haem proteins, iron, phospholipids and endogenous unsaturated lipids that may promote radical formation and hydroperoxide decomposition. Because these constituents entered sequentially while temperature, moisture and oxygen availability also changed, the dominant reaction pathways likely shifted throughout cooking.

Overall, food did not simply increase or suppress lipid degradation. It altered the balance among sterol transformation, triacylglycerol hydrolysis, acylglycerol restructuring, primary oxidation-product formation, hydroperoxide decomposition and secondary carbonyl chemistry. The recovered cooking oils therefore represented the integrated outcome of thermal reactions, oil–food mass transfer, molecular partitioning and interactions with food-derived constituents, explaining why their chemical evolution differed from that of oils subjected to matched heating alone. However, lipids from the food ingredients may also have contributed to changes in the chemical composition of the recovered oils. Since the lipid composition of the meat and other ingredients was not determined in this study, their specific contributions cannot be quantified.

### 3.4. Food–Oil Interactions Reorganize Molecular Association Patterns During Cooking

**Figure 5:**
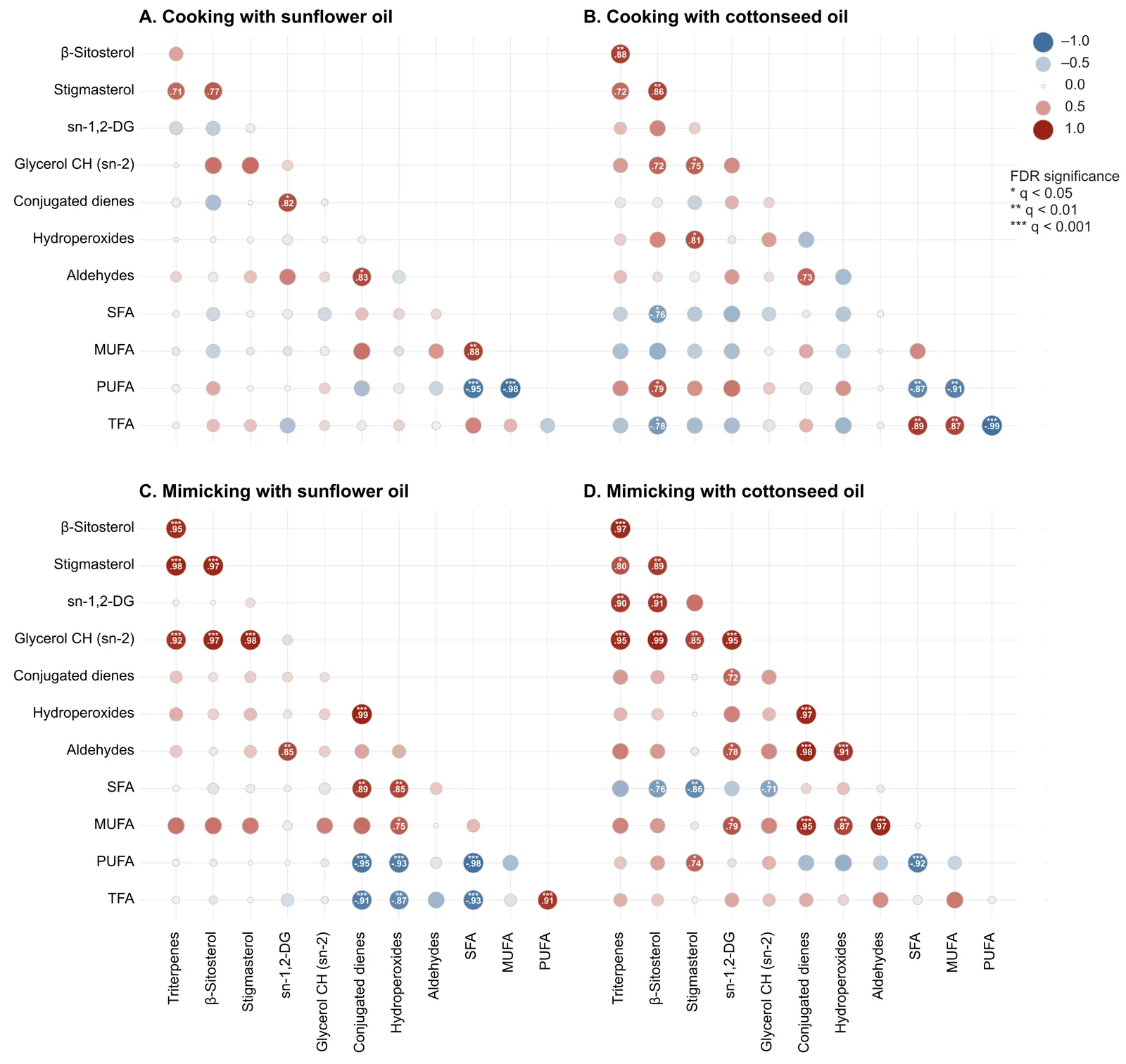
Correlations among fatty-acid classes, minor lipid compounds, hydrolysis products, and oxidation products in sunflower and cottonseed oils during cooking and mimicking. Pearson correlation coefficients were calculated separately for (**A**) sunflower and (**B**) cottonseed oils during cooking, and (**C**) sunflower and (**D**) cottonseed oils during mimicking. Circle colour indicates the direction and magnitude of the correlation coefficient (*r*), ranging from −1 (blue) to +1 (red), while circle size represents the absolute correlation strength. Statistical significance was adjusted for multiple testing using the false discovery rate (FDR).

The preceding sections demonstrated that food modified lipid functional groups (Figure 2), fatty-acid composition (Figure 3), and glyceride remodelling, hydrolysis, and oxidation pathways (Figure 4). Correlation analysis integrates these findings by showing that food altered not only individual molecular constituents but also their relationships, providing a systems-level view of edible-oil transformation during cooking (Figure 5).

Heating alone produced dense and highly coordinated correlation structures in both sunflower and cottonseed oils (Figure 5). Strong positive associations linked sterols, glycerol-backbone resonances, diglycerides, oxidation products and fatty-acid classes, indicating that these variables evolved synchronously during thermal treatment. Such behaviour is expected in relatively simple lipid systems where oxidation, hydrolysis and structural rearrangements are governed mainly by temperature and oxygen availability [1,2].

Cooking fundamentally changed this organisation (Figure 5A,B). Compared with heating, the correlation structures were less densely connected and contained a greater mixture of positive and negative associations, indicating that food progressively uncoupled processes that otherwise evolved coordinately. Rather than remaining a homogeneous lipid phase, the cooking oil became part of a dynamic multiphase system in which oxidation, hydrolysis, lipid migration and structural remodelling proceeded simultaneously but at different rates.

This reorganisation agrees with the analytical observations from Figure 2, Figure 3 and Figure 4. FTIR showed continuous molecular progression during cooking rather than rapid stabilisation after preheating; benchtop NMR demonstrated PUFA depletion accompanied by MUFA and SFA enrichment; and high-field NMR revealed stage-dependent changes in sterols, glyceride structures and oxidation products. Together, these findings indicate that each ingredient addition altered the chemical environment of the oil and redirected its transformation beyond the effect of temperature alone.

The stronger negative associations observed during cooking provide additional mechanistic insight. Inverse relationships between PUFAs and oxidation markers are consistent with the preferential oxidation of polyunsaturated fatty acids, while associations involving sterols may reflect their transformation or redistribution. Relationships between hydroperoxides and aldehydes are compatible with the conversion of unstable primary products into secondary carbonyl compounds, whereas correlations involving diglycerides and glycerol-backbone resonances support continuing hydrolysis and triacylglycerol restructuring. Because correlation does not establish causality, these patterns should be interpreted as coordinated molecular responses rather than direct reaction maps.

The altered correlation structure most likely reflects the progressive increase in system complexity. Sequential addition of onion, meat, carrot, water, and rice changed moisture content, polarity, oxygen availability, and interfacial area while introducing endogenous lipids, proteins, minerals, antioxidants, and pro-oxidants. At the same time, oil entered the food matrix and food-derived lipids and lipophilic compounds migrated into the oil. The abundance of each constituent therefore reflected formation and degradation together with lipid exchange, phase partitioning and reactions with food-derived molecules. Network-based approaches are increasingly used in Foodomics and metabolomics because they reveal coordinated system-level responses that cannot be inferred from individual variables alone.

Overall, the correlation patterns provide independent systems-level evidence supporting the central hypothesis. Heat initiated molecular transformation in both experiments, whereas food–oil interactions reorganised the relationships among fatty-acid remodelling, hydrolysis, sterol transformation, and oxidation during cooking. Edible-oil chemistry under realistic culinary conditions is therefore governed by dynamic interactions with the surrounding food matrix rather than thermal exposure alone.

## 4. Conclusions

This study demonstrates that edible-oil chemistry during realistic cooking cannot be fully explained by modelled heat treatment studies. Although heating initiated molecular transformation, it did not reproduce the progressive changes observed during preparation of Uzbek Palov. Continuous interactions between oil and food instead caused more severe changes in lipid chemistry during cooking, producing greater changes in fatty-acid profiles, glyceride restructuring, and greater transformations in sterols, hydrolysis, and oxidation products. By integrating analysis of oil compositional data from FTIR spectroscopy, benchtop and high-field ^1^H NMR spectroscopy, this study provides a systems-level understanding of edible-oil transformation under realistic culinary conditions. Heat acts as the driving force for lipid transformation, whereas the food matrix determines its molecular outcome through selective oxidation, hydrolysis, lipid exchange, phase partitioning and reactions with food-derived constituents. These findings establish a framework for investigating edible-oil chemistry under realistic cooking conditions rather than simplified heating models. Future studies should characterise both oil and food phases to quantify lipid exchange, identify food-derived modulators of reaction pathways and determine how different matrices reshape oil chemistry. Extending this Foodomics approach to other oils, culinary techniques and food systems will improve interpretation of laboratory heating studies and support healthier and more stable cooking practices.

## Data Availability

The original contributions presented in this study are included in the article/Supplementary Material. Further inquiries can be directed to the corresponding authors.

## Supplementary Materials

The following supporting information can be downloaded at https://www.mdpi.com/article/10.3390/foods15183222/s1, Table S1: Assignment of selected high-field ^1^H NMR spectral regions used for metabolite quantification; Supplementary Method S1: Palov preparation procedure; Figure S1: Joint principal component analysis of sunflower and cottonseed oils during Palov cooking and the corresponding heating-only (mimicking) experiments.
