# Supplementary material for: Molecular Evolution of Sunflower and Cottonseed Oils During Preparation of Uzbek Palov
Source: Foods. 2026 Sep 11;15(18):3222. doi: 10.3390/foods15183222 (PMC13605506; doi:10.3390/foods15183222)

Supplementary Method S1: Palov preparation procedure.

Ingredients

For each independent Palov cooking experiment:

- Edible oil (sunflower or cottonseed): 260 mL
- Onion: 220 g
- Beef chuck: 430 g
- Carrot: 660 g
- Rice: 500 g
- Water: 1100 mL
- Chickpeas: 60 g
- Raisins: 25 g
- Salt: 11 g
- Cumin: 1 g
- Whole black peppercorns: 20 pieces

Cooking and sampling procedure

Step 1 — Prepare ingredients

1. Weigh all ingredients before starting the experiment.
2. Cut the beef chuck into approximately 5 × 5 cm pieces.
3. Cut the carrots into 0.5 × 0.5 cm thick sticks.
4. Slice onions into thin half-rings.
5. Measure 260 mL of sunflower oil or cottonseed oil.
6. Use a 30 cm diameter cast iron pot and a gas heat source.
7. Position a thermometer (50–300 °C range) so that the temperature of the oil phase can be monitored during cooking.

Step 2 — Stage 0: Fresh oil

1. Add 260 mL oil to a 30 cm diameter cast iron pot.
2. Before heating, collect approximately 2 mL oil as the Stage 0 sample.
3. Record the temperature at approximately 22 °C.

Step 3 — Stage 1: Pre-heating

1. Heat the oil using the gas heat source.
2. Adjust the gas flow manually while monitoring the oil temperature.
3. Continue heating for approximately 5–10 min, until the oil reaches 217–275 °C.
4. Collect approximately 2 mL oil as the Stage 1 sample.

Step 4 — Stage 2: Onion addition

1. Add 220 g onion to the heated oil.
2. Fry the onion while stirring with a stainless steel ladle as required.
3. Continue cooking until approximately 14–15 min from the start of the experiment and an oil temperature of 130–145 °C is reached.

4. Collect approximately 2 mL of the recoverable oil phase as the Stage 2 sample.

Step 5 — Stage 3: Meat addition

1. Add 430 g beef to the onion–oil mixture.
2. Continue frying/cooking and stir with a stainless steel ladle as required.
3. Continue until approximately 23–25 min from the start of the experiment and an oil temperature of 115–143 °C is reached.
4. Collect approximately 2 mL of the recoverable oil phase as the Stage 3 sample.

Step 6 — Stage 4: Carrot addition

1. Add 660 g carrot.
2. Continue cooking, stirring as required.
3. Continue until approximately 33–34 min from the start of the experiment and an oil temperature of 102–114 °C is reached.
4. Collect approximately 2 mL of the recoverable oil phase as the Stage 4 sample.

Step 7 — Add water, rice, and remaining ingredients

Add:

- 1100 mL water
- 500 g rice
- 60 g chickpeas
- 25 g raisins
- 11 g salt
- 1 g cumin
- 20 whole black peppercorns

After adding the rice, do not stir the mixture further.

Step 8 — Simmering

1. Cover the cooking vessel with a lid.
2. Reduce/adjust the gas flow manually to maintain the required cooking conditions.
3. Allow the Palov to simmer until approximately 140–143 min from the beginning of the experiment.
4. Maintain the final-stage temperature at approximately 94–100 °C.

Step 9 — Stage 5: Final sampling

1. At approximately 140–143 min, collect approximately 2 mL of the recoverable oil phase.
2. Record this sample as Stage 5.
3. Store the collected oil according to the analytical sample-storage protocol.

Heating-only (Palov-mimicking) experiment

1. Add 260 mL of the corresponding sunflower or cottonseed oil to the same type of cooking vessel.
2. Collect approximately 2 mL of fresh oil as Stage 0.
3. Heat the oil without adding any food ingredients.
4. Manually adjust the gas flow to reproduce the temperature–time profile recorded during the corresponding Palov cooking experiment.
5. Collect approximately 2 mL oil at Stages 1–5 using the same sampling times as in the Palov experiment.
6. No onion, meat, carrot, rice, water, or other ingredients are added during the heating-only experiment.

Table S1. Assignment of selected high-field ^{1}H NMR spectral regions used for metabolite quantification.

Metabolites	Chemical shift (ppm)	Assignment
Triterpenes	0.53–0.58	Methyl protons of triterpene alcohols
β-Sitosterol	0.67–0.69	C18 methyl protons of β-sitosterol
Stigmasterol	0.73–0.78	C18 methyl protons of stigmasterol
sn-1,2-Diglycerides	3.67–3.73	Glycerol backbone of sn-1,2-diglycerides
sn-1,3-Diglycerides	3.95–4.01	Glycerol backbone of sn-1,3-diglycerides
Glycerol CH_2 (sn-1)	4.09–4.17	Glycerol methylene proton at the sn-1 position
Glycerol CH_2 (sn-3)	4.25–4.33	Glycerol methylene proton at the sn-3 position
Glycerol CH (sn-2)	5.23–5.28	Glycerol methine proton at the sn-2 position
Conjugated dienes	5.86–6.40	Conjugated diene protons formed during lipid oxidation
Hydroperoxides	8.31–8.91	Hydroperoxides
Aldehydes	9.45–9.61	Aldehydic protons of secondary oxidation products

Figure S1: Joint principal component analysis of sunflower and cottonseed oils during Palov cooking and the corresponding heating-only (mimicking) experiments.

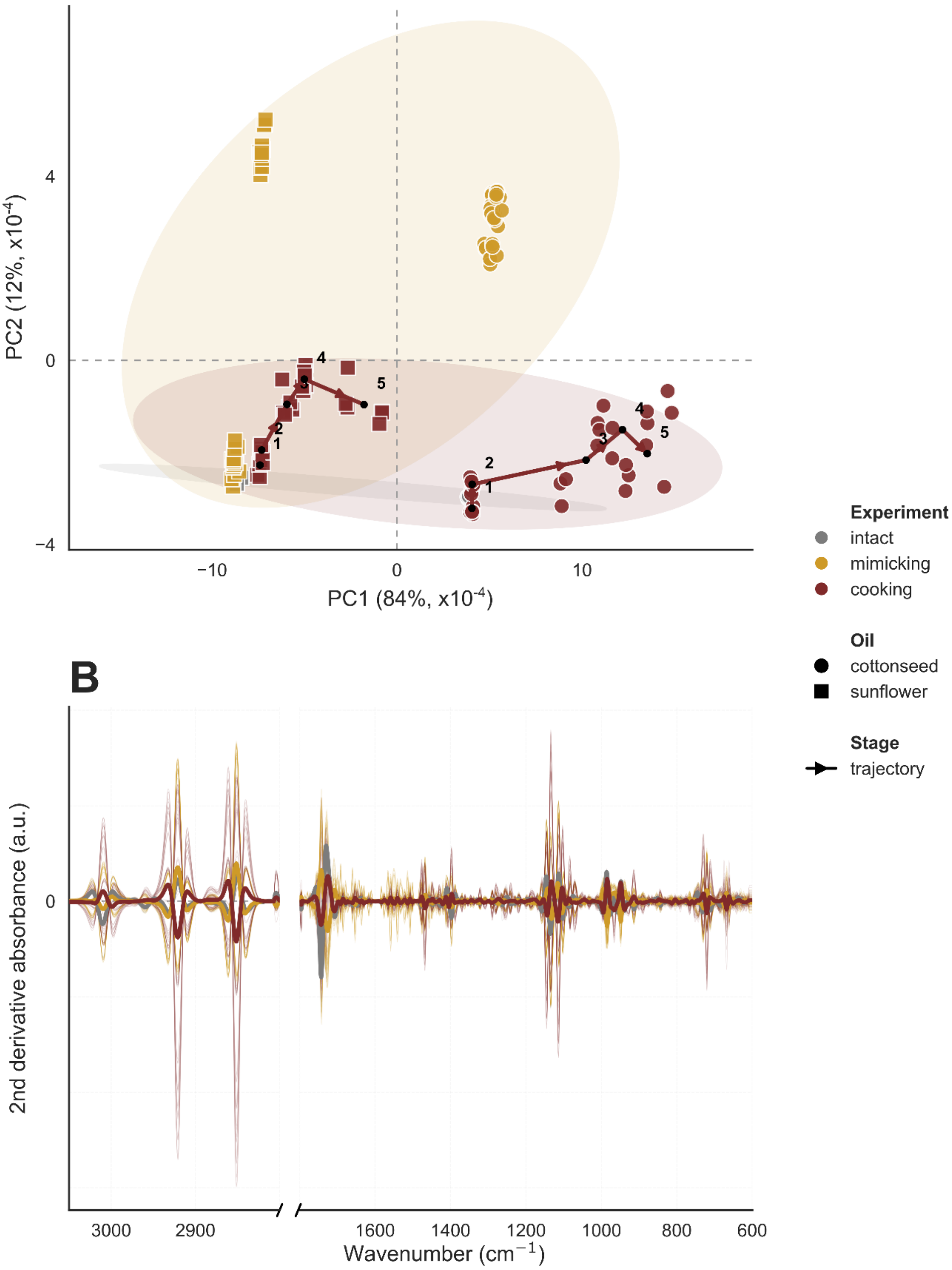

Supplement: Supplementary file 1 [file foods-15-03222-s001.zip › foods-4528986-supplementary.pdf]
